# Exome sequencing identified rare recurrent copy number variants and hereditary breast cancer susceptibility

**DOI:** 10.1371/journal.pgen.1010889

**Published:** 2023-08-14

**Authors:** Timo A. Kumpula, Sandra Vorimo, Taneli T. Mattila, Luke O’Gorman, Galuh Astuti, Anna Tervasmäki, Susanna Koivuluoma, Tiina M. Mattila, Mervi Grip, Robert Winqvist, Outi Kuismin, Jukka Moilanen, Alexander Hoischen, Christian Gilissen, Tuomo Mantere, Katri Pylkäs

**Affiliations:** 1 Laboratory of Cancer Genetics and Tumor Biology, Research Unit of Translational Medicine and Biocenter Oulu, University of Oulu, Oulu, Finland; 2 Department of Pathology, Oulu University Hospital and University of Oulu, Oulu, Finland; 3 Department of Human Genetics and Radboud Institute of Medical Life Sciences, Radboud University Medical Center, Nijmegen, the Netherlands; 4 Department of Surgery, Oulu University Hospital and University of Oulu, Oulu, Finland; 5 Department of Clinical Genetics, Medical Research Center Oulu and PEDEGO Research Unit, Oulu University Hospital and University of Oulu, Oulu, Finland; 6 Department of Internal Medicine and Radboud Center for Infectious Diseases (RCI), Radboud University Medical Center, Nijmegen, the Netherlands; 7 Northern Finland Laboratory Centre Nordlab, Oulu, Finland; National Cancer Institute, UNITED STATES

## Abstract

Copy number variants (CNVs) are a major source of genetic variation and can disrupt genes or affect gene dosage. They are known to be causal or underlie predisposition to various diseases. However, the role of CNVs in inherited breast cancer susceptibility has not been thoroughly investigated. To address this, we performed whole-exome sequencing based analysis of rare CNVs in 98 high-risk Northern Finnish breast cancer cases. After filtering, selected candidate alleles were validated and characterized with a combination of orthogonal methods, including PCR-based approaches, optical genome mapping and long-read sequencing. This revealed three recurrent alterations: a 31 kb deletion co-occurring with a retrotransposon insertion (delins) in *RAD52*, a 13.4 kb deletion in *HSD17B14* and a 64 kb partial duplication of *RAD51C*. Notably, all these genes encode proteins involved in pathways previously identified as essential for breast cancer development. Variants were genotyped in geographically matched cases and controls (altogether 278 hereditary and 1983 unselected breast cancer cases, and 1229 controls). The *RAD52* delins and *HSD17B14* deletion both showed significant enrichment among cases with indications of hereditary disease susceptibility. *RAD52* delins was identified in 7/278 cases (2.5%, P = 0.034, OR = 2.86, 95% CI = 1.10–7.45) and *HSD17B14* deletion in 8/278 cases (2.9%, P = 0.014, OR = 3.28, 95% CI = 1.31–8.23), the frequency of both variants in the controls being 11/1229 (0.9%). This suggests a role for *RAD52* and *HSD17B14* in hereditary breast cancer susceptibility. The *RAD51C* duplication was very rare, identified only in 2/278 of hereditary cases and 2/1229 controls (P = 0.157, OR = 4.45, 95% CI = 0.62–31.70). The identification of recurrent CNVs in these genes, and especially the relatively high frequency of *RAD52* and *HSD17B14* alterations in the Finnish population, highlights the importance of studying CNVs alongside single nucleotide variants when searching for genetic factors underlying hereditary disease predisposition.

## Author summary

For breast cancer susceptibility, CNVs represent an inadequately studied class of genetic variation that could explain a part of the inherited risk. Here, we performed whole-exome sequencing based analysis of rare CNVs in 98 hereditary breast cancer cases and identified recurrent alterations in *RAD52*, *RAD51C* and *HSD17B14* genes. Of these, *RAD52* and *RAD51C* are involved in DNA damage response pathway, whereas *HSD17B14* encodes a protein involved in steroid hormone metabolism. These CNVs were characterized at nucleotide level and genotyped in additional Northern Finnish case-control cohorts. The CNVs that disrupted *RAD52* and *HSD17B14* genes showed enrichment among cases with a family history of the disease and/or early disease onset, suggesting a role for these alterations in breast cancer susceptibility. Overall, the identification of these gene-disrupting recurrent CNVs emphasizes the need to consider CNV detection in the analysis pipelines when searching for novel predisposing alleles for breast cancer.

## Introduction

Breast cancer is the most common cancer in women worldwide accounting for 1 in 4 cancers and for 1 in 6 cancer related deaths [1]. Based on the familial clustering of the disease, it has been estimated that approximately 5–10% of all breast cancers are the result of a strong inherited susceptibility. The well-established breast and/or ovarian cancer susceptibility genes are *BRCA1* and *BRCA2*, along with *PALB2*, *TP53*, *ATM*, *RAD51C* and *CHEK2*. All these genes encode proteins involved in the DNA double-strand break (DSB) signaling pathway and their mutations confer moderate-to-high risk for breast cancer. However, less than half of the familial component of breast cancer is explained by the known susceptibility factors [2]. Resolving this missing component is needed for proper genetic counseling and management of individuals at increased risk of breast cancer, and also for understanding the etiology of this common disease.

For breast cancer susceptibility, copy number variants (CNVs) represent a class of genetic variation that has been poorly studied and could therefore explain at least a part of the inherited risk for the disease. CNVs are generally defined as losses or gains of genomic segments with a size of ≥1 kilobase (kb) and they constitute a major source of genetic diversity in humans [3–5]. For *BRCA1* and *BRCA2*, rare CNVs are estimated to account for ~10% of all the pathogenic mutations, but the CNV prevalence may be higher in populations with founder mutations [6,7]. For example, in the Netherlands, up to one-third of pathogenic *BRCA1* mutations consist of three founder CNV deletions [8]. In addition, a recent CNV analysis showed that 0.5% of unselected breast cancer cases had a CNV deletion in one of the established susceptibility genes, the proportion rising to approximately 1% for cases diagnosed under 50 years of age [9]. However, genome-wide investigations of coding CNVs are relatively scarce and have so far been mainly based on chromosomal microarrays with relatively low resolution [9–11].

Current next-generation sequencing (NGS) approaches are highly efficient in detecting single nucleotide variants (SNVs) and small insertions and deletions (indels), and they can also be utilized for the detection of CNVs [12]. To evaluate the role of rare CNVs in breast cancer susceptibility, here we have performed a whole-exome sequencing based CNV analysis for Northern Finnish high-risk breast cancer cases. Investigations performed in the founder populations, such as the Finns, could enhance the chances for novel discoveries because of the higher prevalence of founder variants compared to outbred populations [13]. Our investigation revealed deletion alleles in *HSD17B14* and *RAD52* that showed significant enrichment in the hereditary cohort and could therefore represent novel moderate-risk alleles for breast cancer. The recurrent duplication in *RAD51C* had also a two-fold increased frequency in cases compared to controls, but the difference remained below statistical significance. The results of this study emphasize the need for including CNV detection in the analysis pipelines when searching for novel breast cancer susceptibility alleles.

## Results

### Identification of rare copy number variants from the whole-exome sequenced discovery cohort

Whole-exome sequencing data from 98 *BRCA1/BRCA2/PALB2* founder mutation-negative Northern Finnish breast cancer cases with indications of hereditary disease susceptibility was used for the identification of rare CNVs (Fig A in S1 Text). In total, CNV calling revealed 56,671 CNV calls in 98 cases, which were filtered further to exclude CNVs less than 1000 bp in size, containing only a single exon, and common CNVs reported [14–16]. The analysis focused on recurrent CNVs with at least three identified carriers, but for the established breast and/or ovarian cancer susceptibility genes, *BRCA1*, *BRCA2*, *PALB2*, *TP53*, *ATM*, *RAD51C* and *CHEK2*, all observed CNVs were evaluated further. After the filtering and validation steps, two recurrent deletions in novel candidate genes remained and were subjected to breakpoint analysis: a 31 kb deletion involving exons 2–9 of *RAD52* (in three cases) and a 13.4 kb deletion entailing exons 4 and 5 of *HSD17B14* (in four cases). Of the previously established breast and/or ovarian cancer susceptibility genes, only *RAD51C* had a CNV alteration. This was a 64 kb duplication covering the exons 1–7 detected in two cases, which matched in size and position to that previously reported [17]. The presence of other known susceptibility alleles was investigated in these nine CNV carriers using the SNV calls from whole-exome sequencing [18]. One case with *RAD51C* duplication was found to carry the *ATM* c.7570G>C (p.Ala2524Pro) allele, recently established to confer a high-risk for breast cancer [19], whereas the rest had no other known moderate-to-high risk predisposing alleles.

### CNV characterization and case-control genotyping

#### *RAD52* delins

**Fig 1:**
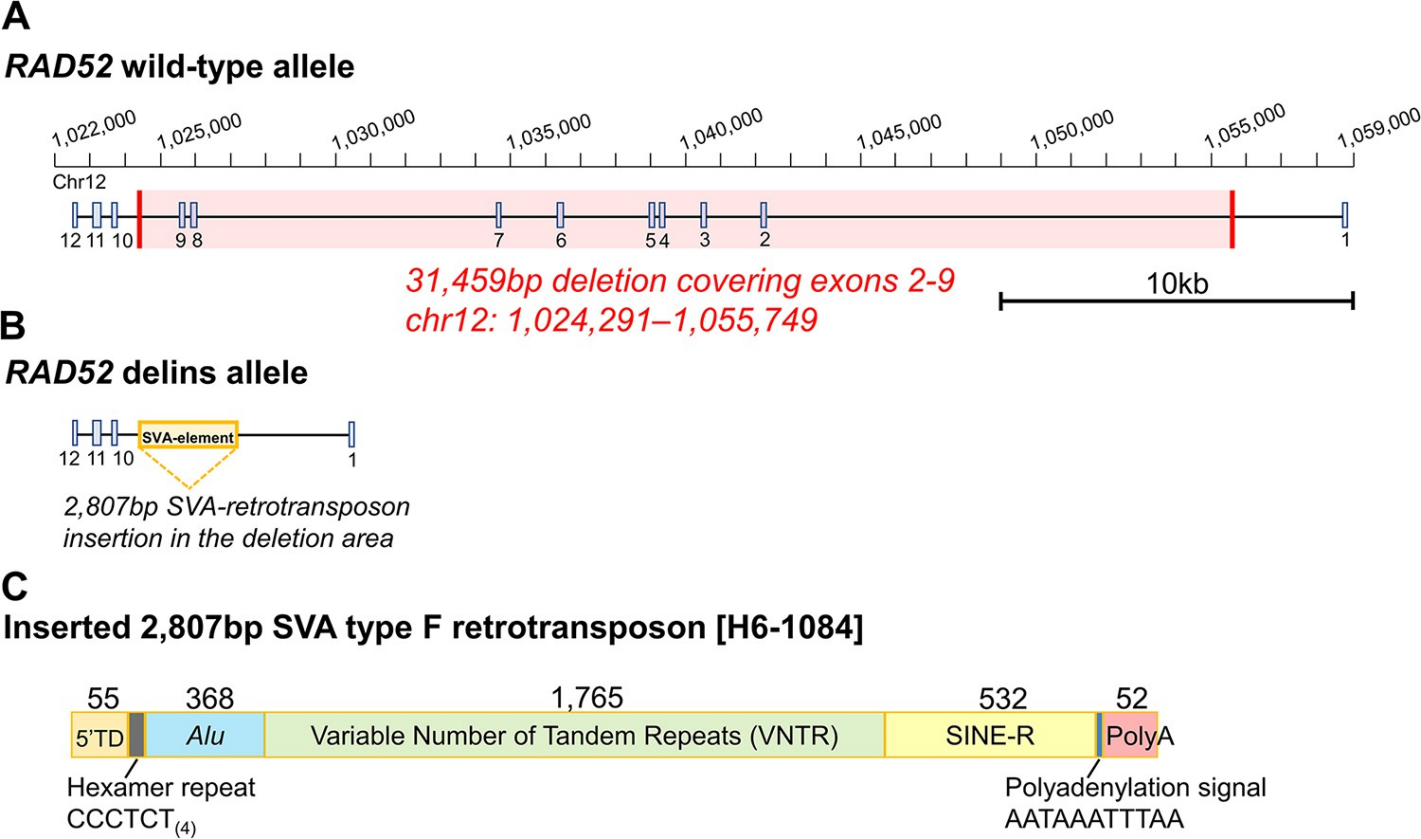
Schematic representation of the *RAD52* delins. (**A**) Wild-type allele with highlighted 31.5 kb deletion covering exons 2–9. (**B**) *RAD52* delins allele, showing the 2.8 kb SVA-transposon insertion into the deletion region. (**C**) Structure of the inserted SVA type F retrotransposon: a transduced sequence (5’ TD), a hexamer CCCTCT_(4)_ repeat, *Alu*-like region, a GC-rich VNTR region, a short interspersed nuclear element (SINE)-R element, polyadenylation signal and a poly-A_(52)_ tail. The length of each region is indicated in base pairs. Exon numbering is based on the *RAD52* transcript NM_134424.4 and the chromosome positions follow build hg19 (GRCh37).

The approximate size and genomic position of the *RAD52* deletion were confirmed with qPCR and optical genome mapping (OGM) (Fig B in S1 Text). The exact breakpoints remained elusive despite multiple attempts by PCR-based approaches. Thus, this alteration was investigated more extensively using long-read whole-genome sequencing in one of the deletion carriers. This revealed that besides deleting 31.5 kb of the genomic region containing *RAD52* exons 2–9, the deletion was accompanied by a 2.8 kb SVA-transposon (SINE-VNTR-ALU) insertion at the breakpoint site (Fig 1). Following the Human Genome Variation Society (HGVS) nomenclature [20], this alteration is annotated as *RAD52* chr12 g.1024291_1055749delins[H6_1084] (hereafter referred as *RAD52* delins). Based on the sequencing results, the deletion had masked the transposon-insertion in OGM analysis, and the extreme GC-content together with the repetitive sequences within the SVA-insertion had rendered the PCR-based approaches unsuccessful. The obtained nucleotide-level information allowed to design PCR-based genotyping assay (Fig 1 and primers in Table A in S2 Text). GnomAD database [21] reports one carrier (European origin) with similar deletion breakpoints (1/10456) but lacks information about the SVA insertion. However, this likely represents the same genomic alteration as described in this study.

The putative association of *RAD52* delins with breast cancer susceptibility was investigated using geographically matched case-control cohorts. It was present in seven breast cancer cases (7/278, 2.5%) from the cohort showing an indication of inherited disease susceptibility (hereafter referred as hereditary cohort, including both the discovery and additional hereditary cohort). Eleven carriers were identified in the controls (11/1229, 0.9%) and this indicated a 2.9-fold increased risk, which is in the 2 to 4-fold range defined for moderate-risk alleles for breast cancer (P = 0.034, odds ratio [OR] = 2.86, 95% confidence interval [CI] = 1.10–7.45, Table 1) [22]. *RAD52* delins carriers from the hereditary cohort were diagnosed with breast cancer at the age of 26, 28, 34, 36, 47, 54 and 78, respectively (mean 43 years), the mean for this cohort otherwise being 45 years (range 26–89 years). In the unselected breast cancer cohort (patients unselected for the family history and age at disease onset), additional twenty *RAD52* delins carriers were identified (20/1983, 1.0%, P = 0.85, OR = 1.13, 95% CI = 0.54–2.36), but this frequency remained similar to that in controls (0.9%). Even though the cases from the hereditary cohort were diagnosed at relatively young age, in the unselected cohort there was no difference in the average age at disease onset between the carriers (mean 60 years, range 50–78 years) and non-carriers (mean 58 years, range 28–93 years). No associations with the clinical parameters or the 5-year breast cancer-specific survival (BCSS) were detected (Table B in S2 Text and Fig C in S1 Text).

**Table 1:** Frequency of *RAD52*, *HSD17B14* and *RAD51C* CNV carriers in the studied breast cancer cases and controls.

Cohort	N	WT	%	Mut^c^	%	OR	95% CI	P^d^
***RAD52* chr12 g.1024291_1055749delins[H6_1084]**								
Hereditary BC^a^	278	271	97.5	7	2.5	2.86	1.10–7.45	0.03
Discovery	98	95	96.9	3	3.1			
Replication	180	176	97.8	4	2.2			
Unselected BC	1983	1963	99.0	20	1.0	1.13	0.54–2.36	0.85
All BC	2261	2234	98.8	27	1.2	1.34	0.66–2.71	0.50
Controls^b^	1229	1218	99.1	11	0.9			
***HSD17B14* chr19 g.49323491_49336860del**								
Hereditary BC^a^	278	270	97.1	8	2.9	3.28	1.31–8.23	0.01
Discovery	98	94	95.9	4	4.1			
Replication	180	176	97.8	4	2.2			
Unselected BC	1983	1959	98.8	24	1.2	1.36	0.66–2.78	0.49
All BC	2261	2229	98.6	32	1.4	1.59	0.80–3.17	0.20
Controls^b^	1229	1218	99.1	11	0.9			
***RAD51C* chr17 c.-31512_965+1210dup{insCTTTTGTGAG}**								
Hereditary BC^a^	278	276	99.3	2	0.7	4.45	0.62–31.70	0.16
Discovery	98	96	98.0	2	2.0			
Replication	180	180	100	0	0			
Unselected BC	1983	1977	99.7	6	0.3	1.86	0.38–9.24	0.72
All BC	2261	2253	99.6	8	0.4	2.18	0.46–10.27	0.51
Controls^b^	1229	1227	99.8	2	0.2			

BC = breast cancer; CI = confidence interval; Mut = variant carrier; OR = odds ratio; WT = wild-type. The chromosome positions follow build hg19 (GRCh37)

^a^ Combined Discovery and Replication cohort

^b^ Northern Finland Red Cross blood donors

^c^ All heterozygous

^d^ Fisher’s exact test, respective case cohort (Hereditary, Unselected, All BC) vs. Controls

Although testing of the family members did not provide strong support for the segregation of *RAD52* delins with breast cancer (Table C in S2 Text), there appeared to be several cases with blood cancers (leukemia, lymphoma or myeloma, 5/27, 18.5%) among the first- or second-degree relatives of the index. Of these, one case with both lymphoma and salivary gland tumor was available for testing and was identified as *RAD52* delins carrier.

#### Prevalence of *RAD52* p.Tyr415Ter (rs4987208) and p.Ser346Ter (rs4987207) variants in the cohorts

Two out of the three *RAD52* delins carriers (Her6 and Her7) identified in the whole-exome sequenced discovery cohort were compound heterozygotes, also carrying either *RAD52* p.Tyr415Ter or p.Ser346Ter, which are polymorphic protein-truncating variants (PTVs) located in the last two exons of *RAD52* (gnomAD). The p.Tyr415Ter variant resides in the last exon and p.Ser346Ter in the second last exon of *RAD52* with minor allele frequencies (MAFs) of 0.0135 and 0.0153 in Finns, respectively (http://www.sisuproject.fi). Due to this observation of compound heterozygosity, all the cases from the hereditary cohort (n = 278) and unselected cohort (n = 1983) were genotyped for these relatively common PTVs (Table D in S2 Text). One additional *RAD52* delins/p.Ser346Ter compound heterozygote was identified (Uns1). In total, 3 out of 27 (11.1%) *RAD52* delins carriers harbored a common PTV in the remaining *RAD52* allele, while none of the 11 delins carriers in the healthy controls were compound heterozygotes. Albeit the observation of compound heterozygotes among affected individuals was interesting, this did not reach statistical significance (P = 0.20, Multilocus Hardy-Weinberg test [23]). Her6, compound heterozygote for *RAD52* p.Tyr415Ter/delins, was diagnosed with ductal, triple-negative breast cancer at the age of 28 years. Curiously, she also carried the *RAD51C* duplication. There was no reported cancer history in the family, and therefore this case could represent an additive model of inheritance. The *RAD52* p.Ser346Ter/delins compound heterozygotes (Her7 and Uns1) were diagnosed with breast cancer at the age of 47 and 50 years, respectively. Her7 had five other breast cancer cases in the family, of which two were confirmed as *RAD52* delins carriers along with one male pancreatic cancer case, but no additional compound heterozygotes were identified. Uns1 had three breast cancer cases in first-degree relatives, but additional samples were not available for testing (Fig D in S1 Text). Altogether, in the hereditary cohort there were fourteen p.Ser346Ter carriers (14/278, 5.0%, P = 0.06, OR = 1.90, 95% CI = 1.01–3.58) and ten p.Tyr415Ter carriers (10/278, 3.6%, P = 0.86, OR = 1.09, 95% CI = 0.54–2.19). In the unselected breast cancer cohort p.Tyr415Ter and p.Ser346Ter frequencies did not differ significantly from the population frequency in this geographical region (Table D in S2 Text). No homozygotes or compound heterozygotes for p.Tyr415Ter and p.Ser346Ter alleles were observed in any of the cohorts.

#### *HSD17B14* deletion CNV

**Fig 2:**
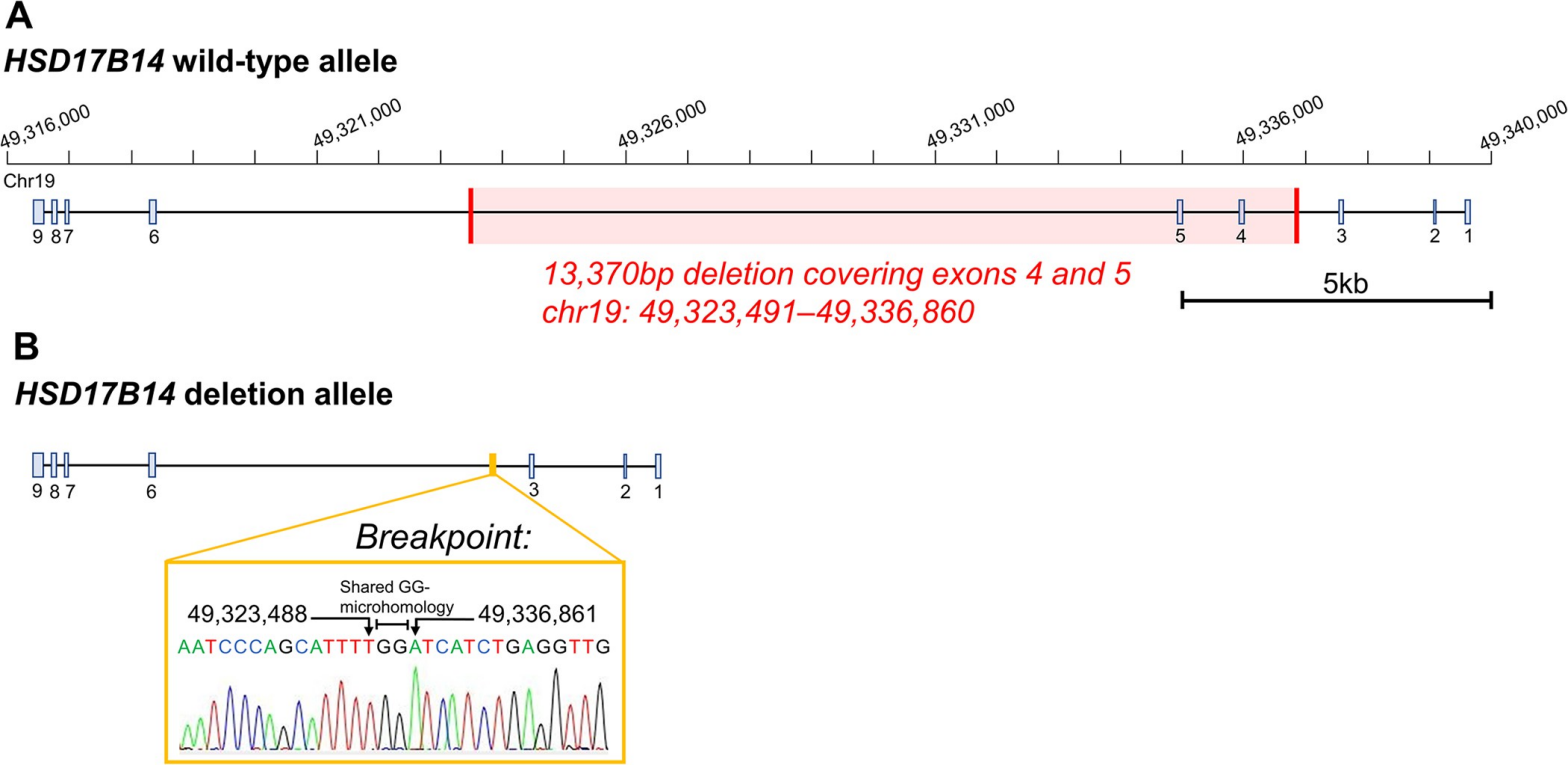
Schematic representation of the *HSD17B14* deletion CNV. (**A**) Wild-type allele with highlighted 13.4 kb deletion covering exons 4 and 5. (**B**) *HSD17B14* deletion allele with a sequence of the deletion breakpoint and its surrounding area. The breakpoint with microhomology is denoted with arrows. Exon numbering is based on the *HSD17B14* transcript NM_016246.3 and the chromosome positions follow build hg19 (GRCh37).

The *HSD17B14* deletion was confirmed by qPCR and OGM, and the precise breakpoints were defined by breakpoint-spanning PCR (primers in Table A in S2 Text) and Sanger sequencing. The deletion (chr19 g.49323491_49336860del) covered the exons 4 and 5 of the gene and was confirmed to be 13.4 kb in size (Fig 2). According to gnomAD database, this *HSD17B14* deletion is rare with only two identified carriers, one in East Asian and one in European cohorts (2/10847, MAF 0.0001).

The *HSD17B14* deletion was detected in altogether eight cases in the hereditary cohort (8/278, 2.9%, P = 0.01, OR = 3.28, 95% CI = 1.31–8.23) and in eleven cases in the control cohort (0.9%, 11/1229), all confirmed as heterozygotes (Table 1). *HSD17B14* deletion carriers from the hereditary cohort were diagnosed with breast cancer at the age of 28, 36, 37, 39, 40, 45, 51 and 56 (mean 42 years). In the unselected cohort, additional 24 carriers were identified (24/1983, 1.2%), but the frequency did not significantly differ from the 11/1229 (0.9%) in controls (P = 0.49, OR = 1.36, 95% CI = 0.66–2.78). The mean age at disease onset for the unselected carriers was 61 years (range 42–80 years), which was similar to the mean of the cohort (58 years). Interestingly, all but one of the *HSD17B14* deletion carrier breast tumors with pathology data available were of Luminal A subtype (21/22, 95%, P = 0.04, OR = 6.52, 95% CI = 0.88–48.63), and all were negative for HER2 amplification (22/22, 100%, P = 0.06, Table E in S2 Text). There was no difference in 5-year BCSS between *HSD17B14* CNV carriers and non-carriers (Fig E in S1 Text). Based on the role of *HSD17B14* in steroid metabolism, we further defined the menopausal status of *HSD17B14* deletion carriers in the unselected breast cancer cohort (pre/post defined by the age at diagnosis). 21 out of 24 carriers (87.5%) were postmenopausal (age at diagnosis 52 years or more), which did not significantly differ from the rest of the unselected cohort (Table F in S2 Text).

The evidence for *HSD17B14* deletion segregating with cancer phenotype within these families remained inconclusive (Table G in S2 Text). However, two *HSD17B14* CNV carriers (Her14 and sister of Uns32, confirmed as carrier) were also diagnosed with polycythemia vera. This myeloproliferative neoplasm is very rare (incidence 1.70 cases per 100,000 [24]) and reported altogether in three individuals (of which two were *HSD17B14* CNV carriers) in the studied breast cancer cohort and their family members. Her14 was diagnosed with breast cancer at the age of 56 and polycythemia vera 6 years later, while the other case (sister of Uns32) was diagnosed with cervical cancer at the age of 68 and polycythemia vera 2 years later.

#### *RAD51C* duplication CNV

The currently identified *RAD51C* duplication was confirmed as the same chr17 c.-31512_965+1210dup{insCTTTTGTGAG} allele that has previously been reported in breast and ovarian cancer cases from a Southern Finnish cohort (6/2533, 0.2%) [17]. It creates an extra copy of the upstream sequence and exons 1–7, which is located 31 kb upstream of the intact copy of the *RAD51C* gene. The duplication is absent from the gnomAD database indicating that it is a rare Finnish founder allele. In the current study, two *RAD51C* duplication carriers were observed in the hereditary cohort (2/278, 0.7%, P = 0.16, OR = 4.45, 95% CI = 0.62–31.70, Table 1), diagnosed with breast cancer at the age of 28 and 44. In the unselected cohort, six additional carriers were identified (6/1983, 0.3%, P = 0.72, OR = 1.86, 95% CI = 0.38–9.24), but the frequency did not differ from that in healthy controls (2/1229, 0.2%). Although in total *RAD51C* CNV had slightly higher frequency in the cases, this remained under statistical significance, even when combined with the previously published results from the Southern Finnish cohort (14/4794, 0.3% in cases with breast cancer vs. 2/2502, 0.1% in controls, P = 0.07, OR = 3.67, 95% CI = 0.83–16.12). The mean age at disease onset for the currently identified unselected carriers was 60 years (range 39–80 years), which was similar to the mean of the cohort (58 years). All six carriers from the unselected cohort with tumor pathology data available had estrogen (ER) and progesterone receptor (PR) positive tumors (Table H in S2 Text). There was no difference in 5-year BCSS between the *RAD51C* CNV carriers and non-carriers (Fig F in S1 Text). Even though this CNV has previously been associated with increased ovarian cancer risk [17], only one of the families in our cohort had reported an ovarian cancer case, but no DNA sample was available for testing (Table I in S2 Text).

Interestingly, two cases with *RAD51C* duplication were double carriers of the currently investigated CNVs: Her6 (breast cancer at 28 years) had both the *RAD51C* duplication and *RAD52* delins/Tyr415Ter variants, while Uns38 (breast cancer at 65 years) had both the *RAD51C* duplication and *HSD17B14* deletion. No double carriers for any of the currently studied CNVs were identified in the controls.

## Discussion

Genome-wide investigation of rare coding region CNVs in 98 high-risk Northern Finnish breast cancer cases revealed recurrent alterations in *RAD51C*, *RAD52* and *HSD17B14* genes. Of these, *RAD51C* duplication has previously been described [17], whereas the *RAD52* delins and *HSD17B14* deletion CNVs were characterized and investigated here for the first time for their association with breast cancer susceptibility.

*RAD51C* mutations have previously been reported to increase the risk for breast and ovarian cancer and to also be causative for a recessively inherited Fanconi anemia-like disorder [25], similar to *BRCA1*, *BRCA2* and *PALB2* mutations. These genes are important players in the DNA DSB repair pathway through homologous recombination (HR) [2,26]. HR-deficiency has been shown to be common in breast cancer, and over 20% of all cases show a HR-deficiency induced mutational signature in their tumors [27]. The relative risk caused by *RAD51C* mutations has been estimated higher for ovarian cancer (risk ratio 7.55) than it is for breast cancer (risk ratio 1.99), which places *RAD51C* in the category of moderate-risk genes [28]. In the current study the *RAD51C* duplication CNV allele had two-fold frequency in the breast cancer cases compared to controls. However, potentially due to its rarity this remained under statistical significance, even when combined with the results from the Southern Finnish cohort. We found no supporting evidence for increased ovarian cancer risk, as there was only one reported ovarian cancer case in the eight currently identified carrier families. Pathogenic *RAD51C* variants have previously also been linked particularly to ER-negative and triple-negative breast cancer subtypes [29,30], but all six identified carriers from the unselected cohort turned out to be ER- and PR-positive. It is likely that while the currently observed duplication is a large-scale genomic event, two intact copies of the gene remain, thereby having a different effect at the functional level than loss-of-function alleles.

*RAD52* also encodes a protein involved in the DSB repair pathway with best-defined role as a key mediator of the repair by single-strand annealing (SSA) [31]. SSA repair is one of the three competing pathways (HR, SSA and alternative end joining) to repair DSB, but due to the mutagenic nature of the SSA and alternative end joining pathways, cells favor HR pathway whenever possible [32]. RAD52 also seems to have a role in HR as it can substitute BRCA2 function in loading RAD51 onto the resected single-stranded DNA, albeit at a lower efficiency [33]. In human cell cultures, co-knockdown of *RAD52* and any of the genes from the *BRCA1/BRCA2/PALB2* axis is lethal and shows significant reduction in *RAD51* foci [34].

*RAD52* harbors two polymorphic PTV alleles, p.Ser346Ter and p.Tyr415Ter, both located at the 3’ end of the gene (gnomAD). Of these, p.Ser346Ter has previously been investigated in the context of *BRCA1*/*BRCA2* mutations. In pathogenic *BRCA2* and to lesser extent *BRCA1* germline mutation carriers, the presence of *RAD52* p.Ser346Ter was associated with reduced risk of breast cancer [35]. Since p.Ser346Ter PTV localizes to the end of the gene, the mutant protein retains all the functional domains and is likely functional. However, it loses the last eight amino acids that contain the nuclear localization signal leading to protein localization predominantly in the cytoplasm, which weakens its ability to influence HR or SSA [36]. In contrast to the polymorphic PTVs, the currently observed *RAD52* delins abolishes a large part of the gene and is most likely a null allele. Large loss-of-function deletions in *RAD52* are very rare according to public databases (gnomAD), and the currently described is the first one reported with nearly 1% carrier frequency in general population in Northern Finland and 2.5% occurrence in cases with an indication of inherited breast cancer predisposition. As the two of the identified *RAD52* delins carriers were compound heterozygotes for p.Ser346Ter, and one for p.Tyr415Ter (altogether 3/27, 11%), this suggests potential additive effects for the observed *RAD52* alterations in breast cancer predisposition. To support this, no compound heterozygotes were observed in the controls.

Long-read whole-genome sequencing revealed that the *RAD52* deletion co-occurred with an insertion, which exhibited 99% homology to the retrotransposon SVA_F element H6_1084. Similar insertions have also been reported to be involved in pathogenic deletions of *NF1* in neurofibromatosis [37]. Another recent study described two siblings with atypical teratoid rhabdoid tumors as having a pathogenic SVA-transposon insertion in *SMARCB1* [38]. As the SVA-transposon insertions are challenging to detect when using short-read based NGS technologies, and as active SVA elements are pervasive in the human genome, it has been suggested that these could present recurrent and less-studied mechanism causing CNV deletions [39]. For this hypothesis, the current study provides further support. It is also noteworthy that such insertions accompanying deletions may cause PCR-based validation approaches to fail.

The third recurrent CNV was detected in *HSD17B14* (17β-hydroxysteroid dehydrogenase), which encodes an enzyme involved in steroid metabolism and converting intracellular 17β-estradiol into estrone in human tissues, including mammary fat [40]. As a member of large HSD17B enzyme family the exact roles of the gene are not well-documented. However, elevated *HSD17B14* expression has been associated with poor survival in women with ER-positive breast cancer, and a high estrone (E1):17β-estradiol (E2) ratio in postmenopausal women has been shown to drive inflammation and stimulate ER-positive breast cancer tumor growth [41]. Consequently, it has also been suggested that proper balance of estrogens (E1:E2 ratio), in which HSD17B14 enzyme plays a critical role, could be crucial for breast cancer growth and metastasis [42]. Of the currently identified *HSD17B14* deletion carriers with available tumor data from the unselected cohort, all but one had ER-positive tumors, but the menopausal status did not significantly differ compared to the rest of the unselected cohort. The *HSD17B14* deletion was most prevalent in the hereditary cohort, and the risk is estimated as moderate with 3-fold enrichment compared to controls. This CNV is one of the two predicted loss-of-function *HSD17B14* CNV alleles reported in the gnomAD database. Although rare, they could represent alleles related to breast cancer predisposition in other populations as well, warranting further investigations.

Overall, the identification of recurrent deletions in *RAD52* and *HSD17B14* genes, along with previously described duplication in *RAD51C*, suggests that rare CNVs contribute to increased breast cancer risk, potentially in combination with other moderate-risk alleles as indicated by the identification of several double-mutants. As the current discovery cohort was limited in sample size, it is possible that additional causal CNVs exist, warranting studies in larger cohorts. The combined prevalence for the *RAD52* and *HSD17B14* alleles in the hereditary breast cancer cohort was as high as 5%. Whereas *RAD51C* is a previously established cancer susceptibility gene, both *RAD52* and *HSD17B14* also encode proteins involved in pathways previously identified as essential for breast cancer development: the DNA damage response pathway is frequently aberrated in breast cancer and strongly linked to inherited predisposition, whereas steroid hormone metabolism can drive breast cancer progression. The current results highlight the need for careful examination of CNVs alongside SNVs and small indels when searching for new breast cancer risk factors, and when evaluating the potential combined effects of moderate-risk alleles.

## Materials and methods

### Ethics statement

The study was approved by the Ethics Committee of the Northern Ostrobothnia Hospital District (100/2016, amendments 2021, 2022) with informed consent of the participants (verbal for hereditary cases collected before 2000, written for all others). The study was conducted in compliance with the Declaration of Helsinki.

### CNV calling with ExomeDepth from the discovery cohort

Whole-exome sequencing data from 98 *BRCA1/BRCA2/PALB2* founder mutation-negative Northern Finnish breast cancer cases with indications of hereditary disease susceptibility was used for CNV calling. This discovery cohort consisted of: 1) index cases from families with ≥3 breast and/or ovarian cancer cases in first- or second-degree relatives (n = 83), 2) index cases from families with two cases of breast, or breast and ovarian cancer in first- or second-degree relatives, of which at least one with early disease onset (<35 years), bilateral breast cancer or multiple primary tumors (n = 7) and 3) breast cancer cases diagnosed at or below the age of 40 (n = 8). The early-onset breast cancer cases were included based on the assumption that when a woman below the age of 40 years develops breast cancer, a hereditary predisposition can be suspected regardless of the family history [43]. Whole-exome library preparation, sequencing and annotation have been described in detail in Koivuluoma et al. 2021, where the same dataset was used to detect predictably deleterious rare SNVs and small indels [18].

CNVs were called with ExomeDepth algorithm, which is based on counting the reads aligned to the coding areas of the genome [44]. CNV calls were further filtered as follows: 1) excluding CNVs <1000 bp in size, 2) excluding variants with observed vs. expected reads ratio >0.6 for deletions and <1.4 for duplications, 3) excluding CNVs containing only a single exon, 4) excluding common CNVs reported in Conrad et al. 2010 [14] and 5) excluding CNVs with MAF >1% in Database of Genomic Variants or Bionano Genomics control database [15,16]. CNVs selected for further characterization in novel candidate genes had to be recurrent with at least three identified carriers in the discovery cohort. For the established breast and/or ovarian cancer susceptibility genes, *BRCA1*, *BRCA2*, *PALB2*, *TP53*, *ATM*, *RAD51C* and *CHEK2*, all the observed putative CNVs were evaluated further (Fig A in S1 Text).

The CNV alleles selected for further analysis were confirmed with genomic qPCR as described in Pylkäs et al. 2012 [11] and subjected to breakpoint characterization using a combination of breakpoint-spanning PCR, OGM and/or PacBio HiFi long-read genome sequencing.

### Optical genome mapping

OGM was performed as described in Mantere et al. 2021 [45] to confirm the approximate size and genomic position of the observed CNV alleles (*RAD51C*, *RAD52*, *HSD17B14*) in three carriers. Briefly, ultra-high molecular weight (UHMW) gDNA was isolated from frozen EDTA blood, following the manufacturer’s guidelines (Bionano Prep SP Frozen Blood DNA Isolation Protocol, Bionano Genomics #30395). For each sample, 750 ng of purified UHMW gDNA was labeled with DL-green fluorophores with the Direct Labeling Enzyme (DLE-1) chemistry and cleaned up with membrane adsorption (Bionano Prep Direct Label and Stain Protocol, Bionano Genomics, #30206). Labeled gDNA samples were loaded on the Saphyr chips for linearization and imaging on the Saphyr instrument. 800 Gbp of data was collected per sample using GRCh38/hg38 as a reference. The *de novo* assembly and SV annotation pipeline were executed with Bionano Solve software Solve3.7 using Access v1.7 (Bionano genomics).

### PacBio long-read genome sequencing

*RAD52* and *RAD51C* CNV alleles were characterized at nucleotide level using HiFi long-read genome sequencing as described in Sabatella et al. 2021 using the Sequel II system (Pacific Biosciences) [38]. Briefly, the SMRTbell libraries were prepared according to Procedure & Checklist–Preparing HiFi SMRTbell Libraries using SMRTbell Express Template Prep Kit 2.0 (Pacific Biosciences). Following the sequencing, circular consensus reads were analyzed and mapped in SMRTlink 8.0 against human reference GRCh38/hg38. Structural variants were called using pbsv v2.2.2 (SMRTLink v8.0.0) and annotated using an in-house pipeline with public databases [38].

### Genotyping of case-control cohorts

The association of the three recurrent CNVs with breast cancer susceptibility was investigated using geographically matched case-control cohorts. Additional breast cancer cases with an indication of inherited predisposition were used as a replication cohort and consisted of 180 *BRCA1*, *BRCA2*, *PALB2* and *MCPH1* founder mutation negative [46–48] index cases from breast cancer families (n = 129) or cases with early-onset (≤40 years) breast cancer (n = 51). Familial index cases were from families with 1) three or more breast and/or ovarian cancer cases in first- or second-degree relatives (n = 56), 2) two cases of breast, or breast and ovarian cancer in first- or second-degree relatives, of which at least one with early disease onset (<35 years), bilateral disease or multiple tumors (n = 37) and 3) two cases of breast cancer in first- or second-degree relatives (n = 36).

Unselected breast cancer cohort included 1983 consecutive Northern Finnish cases diagnosed at the Oulu University Hospital during the years 2000–2019 and were unselected for the family history of cancer and age at disease onset. The mutational status for known predisposing gene mutations was either unknown or not used as an exclusion criterion for this cohort. Clinical parameters of the breast tumors were obtained from pathology reports. Geographically matched anonymous, cancer-free Northern Finnish Red Cross blood donors (n = 1229) were used as population controls (*n* = 759 females and *n* = 470 males).

CNV genotyping was performed using DNA extracted from peripheral blood by multiplex high-resolution melt (HRM) analysis (CFX96, Bio-Rad, and Type-IT HRM reagents, Qiagen). For each CNV, breakpoint-spanning allele-specific PCR primers together with control PCR reaction was used. Samples with verified CNVs were used as positive controls. All the positive samples from PCR-based genotyping were confirmed with Sanger sequencing (ABI3500xL Genetic Analyzer, Applied Biosystems), and the zygosity of the deletion alleles was defined by separate wild-type specific PCR. In the segregation analysis, formalin-fixed paraffin-embedded (FFPE) tissue samples from relatives were genotyped using shorter amplicons and direct sequencing. The genotyping of the polymorphic 3´ end PTVs in *RAD52* (rs4987207 and rs4987208) was performed with HRM analysis and iPLEX (Agena Bioscience MassARRAY System, Sequenom Inc., FIMM). All primers used for genotyping and sequencing are presented in Table A in S2 Text.

### Statistical analyses

Statistical analyses were performed with IBM SPSS Statistics 26.0 (IBM Corporation). Mutation carrier frequencies between cases and controls, and differences in the tumor characteristics between carrier and non-carrier cases were both compared with Fisher’s exact test. The mean age at diagnosis between carriers and non-carriers in the unselected cohort was compared with Mann-Whitney U test. The potential enrichment of *RAD52* PTVs (stop-gain variants rs4987207 and rs4987208) in the *RAD52* delins carriers was evaluated with Hardy-Weinberg Multilocus analysis [23]. The 5-year BCSS between the CNV carriers and non-carriers from the unselected breast cancer cohort was compared by univariate Kaplan-Meier analysis and Cox regression. The time from date of diagnosis to the last follow-up or death was calculated as survival time. All p-values were two-sided and values ≤0.05 were considered statistically significant.

## Supporting information

S1 Text Supporting Figs A-F. (DOCX)

S2 Text Supporting Tables A-I. (DOCX)
