# Supplementary material for: Exome sequencing identified rare recurrent copy number variants and hereditary breast cancer susceptibility
Source: PLoS Genet. 2023 Aug 14;19(8):e1010889. doi: 10.1371/journal.pgen.1010889 (PMC10449128; doi:10.1371/journal.pgen.1010889)
Supplement: S1 Text — (DOCX) [file pgen.1010889.s001.docx]

**
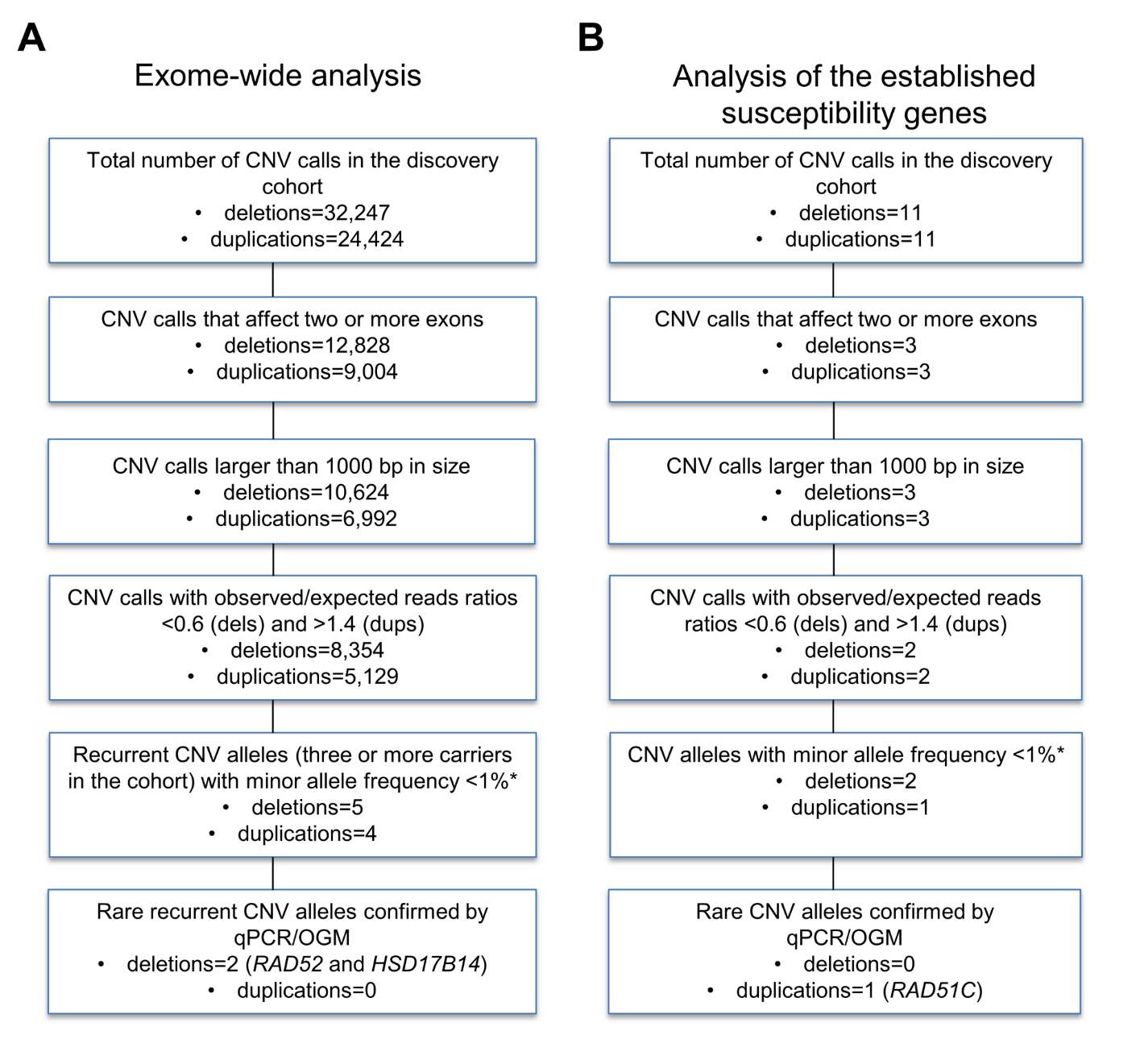
**

**Fig A. Filtering steps for the ExomeDepth CNV data.** (**A**) Filtering steps for all observed CNV calls from the whole-exome sequenced 98 discovery cohort cases, (**B**) Filtering steps for the established breast and/or ovarian cancer susceptibility genes *BRCA1*, *BRCA2, PALB2*, *TP53*, *ATM*, *RAD51C* and *CHEK2.*

*Excluding CNV alleles present in Conrad et al. 2010 reporting common CNVs, and/or with minor allele frequency >1% in Database of Genomic Variants or Bionano Genomics control database

**
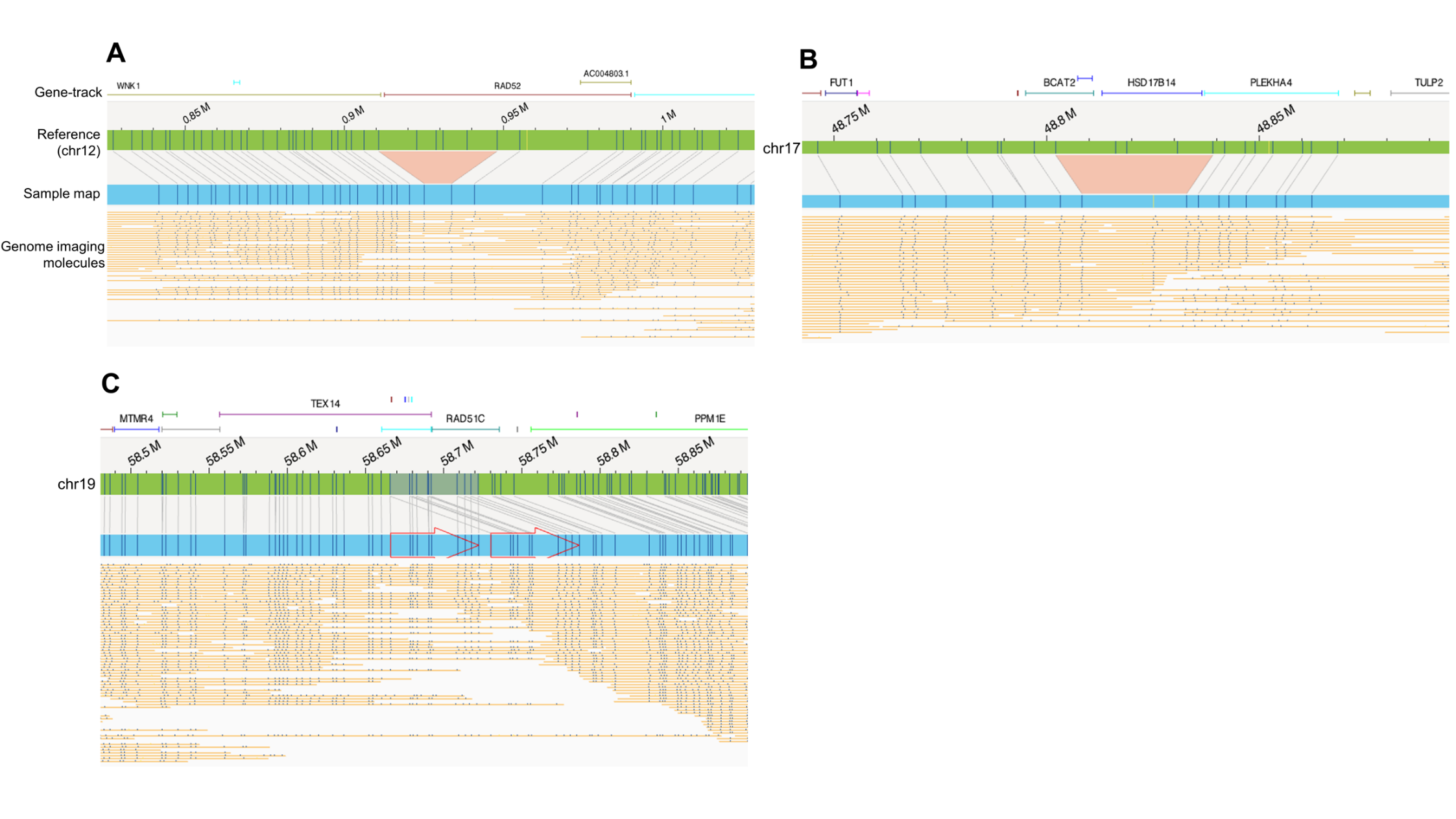
**

**Fig B. Copy number variant confirmation by optical genome mapping.** Single-molecules (yellow lines) are *de novo* assembled into genome maps (blue bar) and aligned to the reference (green bar, Hg38). Name and location of the genes are shown above the reference in the gene-track. Red markings indicate the approximate size and position of the CNVs. (**A**) Predicted 28,640bp deletion in *RAD52*. (**B**) Predicted 12,337bp deletion in *HSD17B14*. (**C**) Predicted 56,394bp tandem duplication in *RAD51C*.


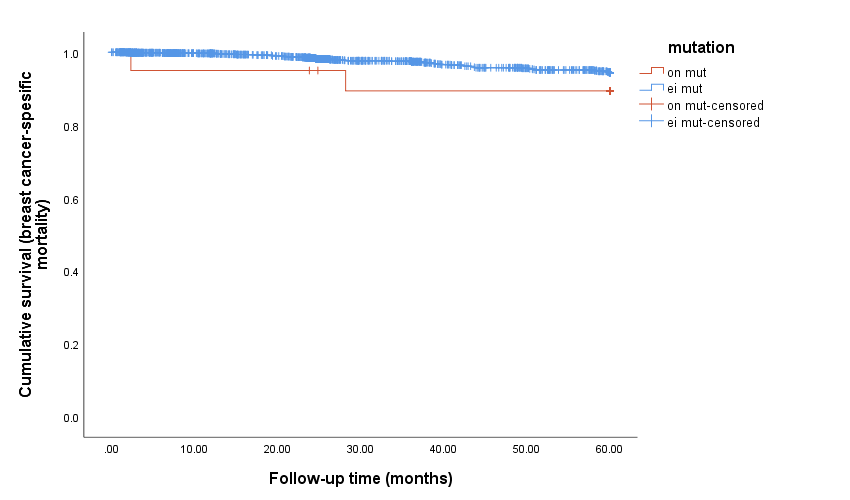


Cox regression
Hazard ratio=2.08
95% confidence interval =0.5-8.5
P=0.31

**Cumulative survival (breast cancer-specific mortality)**

**Follow-up time (months)**

Log-rank p=0.301

*RAD52* delins

wild-type
carrier

**Fig C. The 5-year breast cancer specific survival of *RAD52* delins carriers and non-carriers (wild-type).**


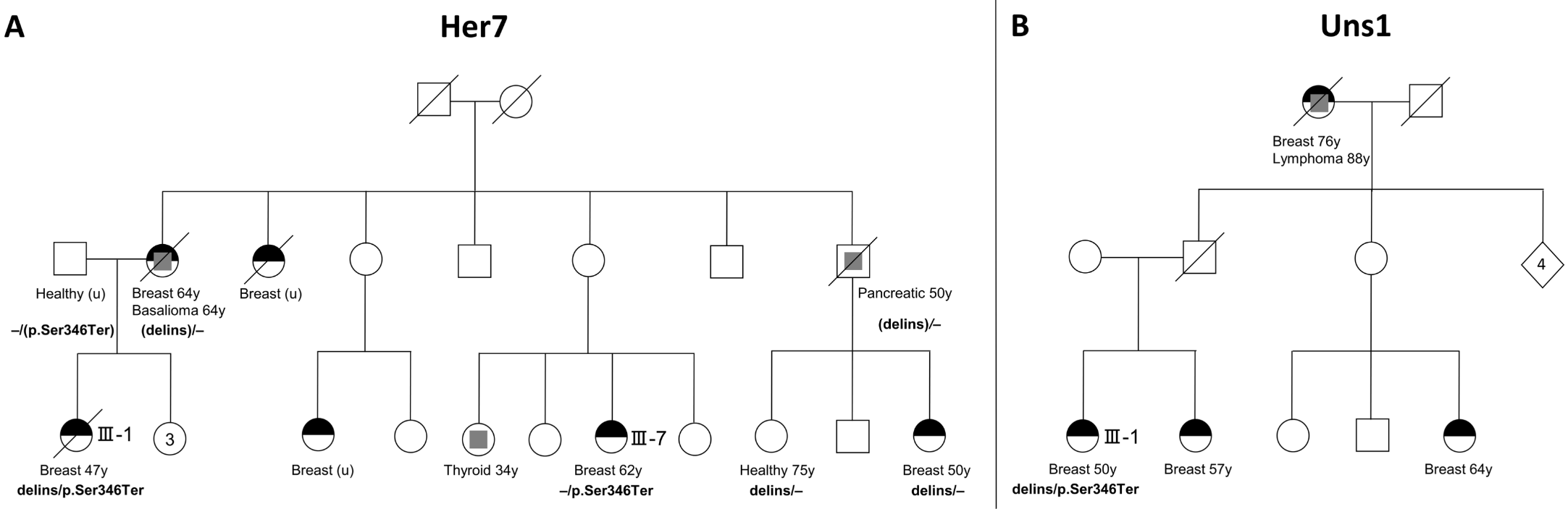


**Fig D. Pedigrees of the *RAD52* delins and p.Ser346Ter compound heterozygotes.** (**A**) Her7 (III-1) family. The inheritance pattern for p.Ser346Ter variant in cousin (III-7) is unclear. However, *RAD52* delins and Ser346Ter alterations have been confirmed to reside in different haplotypes with PacBio long-read sequencing. (**B**) Uns1 (III-1) family. Obligatory carriers are marked with parenthesis. y=years; u=age at diagnosis/follow-up unknown.


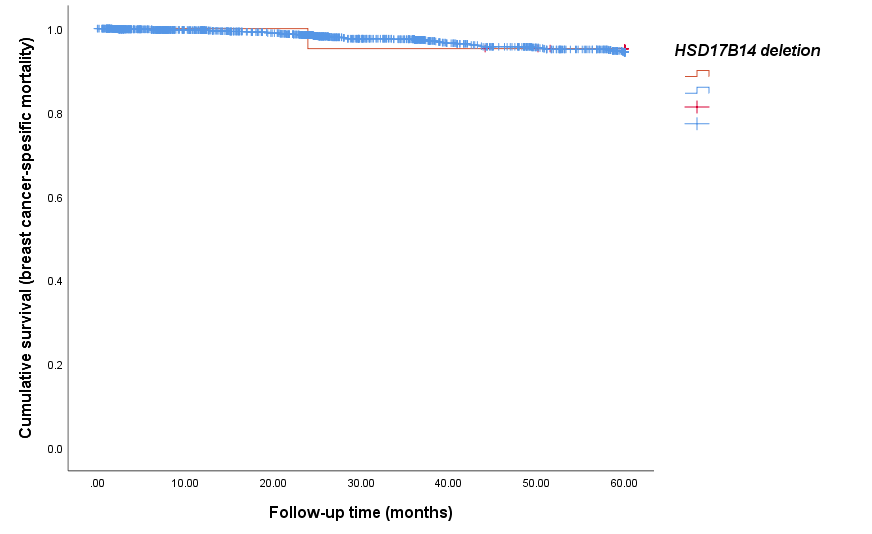


Cox regression
Hazard ratio=0.87
95% confidence interval=0.1-6.3
P=0.87

**Cumulative survival (breast cancer-specific mortality)**

**Follow-up time (months)**

Log-rank p=0.894

*HSD17B14* deletion

wild-type
carrier

**Fig E. The 5-year breast cancer specific survival of *HSD17B14* deletion carriers and non-carriers (wild-type).**


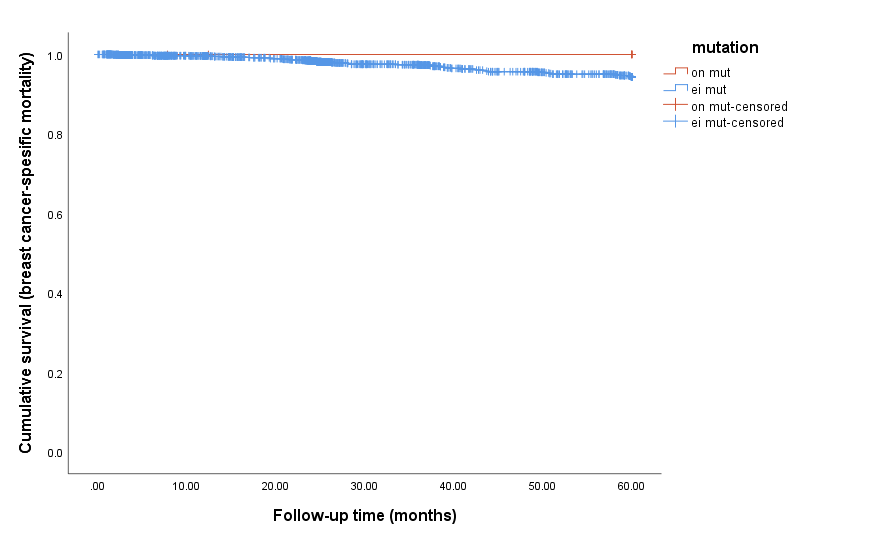


*RAD51C* duplication

Log-rank p=0.625

Cox regression
Hazard ratio=0.05
95% confidence interval=0-3313466
P=0.74

wild-type
carrier

**Follow-up time (months)**

**Cumulative survival (breast cancer-specific mortality)**

**Fig F. The 5-year breast cancer specific survival of *RAD51C* duplication carriers and non-carriers (wild-type).**
