# Supplementary material for: Exome sequencing identified rare recurrent copy number variants and hereditary breast cancer susceptibility
Source: PLoS Genet. 2023 Aug 14;19(8):e1010889. doi: 10.1371/journal.pgen.1010889 (PMC10449128; doi:10.1371/journal.pgen.1010889)
Supplement: S2 Text — (DOCX) [file pgen.1010889.s002.docx]

**Table A. Primers used for the CNV breakpoint spanning PCRs and *RAD52* stop-gain variant genotyping**

| Primer designation | Assay | Sequence (5’ - 3’) |
| --- | --- | --- |
| *RAD52_*delins*_*Forward | CNV breakpoint spanning PCR | GTCTGGCTCGCTTGTTACAG |
| *RAD52_*delins*_*Reverse (binds to the inserted SVA element) | CNV breakpoint spanning PCR | GATCACTCGCGGTTAGGGG |
| *HSD17B14_*deletion*_*Forward | CNV breakpoint spanning PCR | AACAAACCGAAAAAGAACGTT |
| *HSD17B14_*deletion*_*Reverse | CNV breakpoint spanning PCR | GTGGGTCAACTGGAACTGATT |
| *RAD51C_*duplication*_*Forward | CNV breakpoint spanning PCR | TTACTATTGACTGTCGCCCC |
| *RAD51C_*duplication*_*Reverse (binds on top of the breakpoint) | CNV breakpoint spanning PCR | TGGCATCTCAACTCACAAAAGG |
| *HSD17B14_*FFPE*_*Forward | FFPE-sample genotyping | AACAAACCGAAAAAGAACGTT |
| *HSD17B14_*FFPE*_*Reverse | FFPE-sample genotyping | CCAACCTCAGATGATCCAAA |
| *RAD52*_rs4987207_Forward | p.Ser346Ter stop-gain variant (rs4987207) genotyping | CTTGTGGTTTCTGGTGGCAA |
| *RAD52*_rs4987207_Reverse | p.Ser346Ter stop-gain variant (rs4987207) genotyping | GCTGATGACGCACTTTTCCT |
| *RAD52*_rs4987208_Forward | p.Tyr415Ter stop-gain variant (rs4987208) genotyping | TCCCTTTGTGACAGAGTCCA |
| *RAD52*_rs4987208_Reverse | p.Tyr415Ter stop-gain variant (rs4987208) genotyping | TCTGCCTTCCTCCTAACAGT |
| *RAD52*_Control_Forward | CNV PCR control amplicon | GTCTGGCTCGCTTGTTACAG |
| *RAD52*_Control_Reverse | CNV PCR control amplicon | ACGGGAACTTTTGGCAGATT |
| *HSD17B14*_Control_Forward | CNV PCR control amplicon | TGACGTTCATTCTCCAGCAG |
| *HSD17B14*_Control_Reverse | CNV PCR control amplicon | AGCAGAGAGGTGTTTAGGCA |
| *RAD51C*_Control_Forward | CNV PCR control amplicon | TTACTATTGACTGTCGCCCC |
| *RAD51C*_Control_Reverse | CNV PCR control amplicon | AGGCAAACTTTTCCTCCTGT |

**Table B. Tumor parameters of unselected cohort *RAD52* delins carriers compared with the tumors of non‐carriers**

| Category | Mut | % | WT | % | P^b^ | OR | 95% CI |
| --- | --- | --- | --- | --- | --- | --- | --- |
| T class |  |  |  |  |  |  |  |
| 1 | 12 | 66.7 | 1089 | 64.0 | 1 | 1.12 | 0.42–3.01 |
| 2 | 5 | 27.8 | 537 | 31.6 | (1 vs. 2, 3, 4) |  |  |
| 3 | 1 | 5.6 | 54 | 3.2 |  |  |  |
| 4 | 0 | 0 | 21 | 1.2 |  |  |  |
| M class |  |  |  |  |  |  |  |
| Negative | 19 | 95.0 | 1915 | 97.8 | 0.36 | 0.43 | 0.06–3.26 |
| Positive | 1 | 5.0 | 43 | 2.2 |  |  |  |
| Node status |  |  |  |  |  |  |  |
| Negative | 13 | 65 | 1262 | 64.8 | 1 | 1.01 | 0.40–2.54 |
| Positive | 7 | 35 | 686 | 35.2 |  |  |  |
| ER status |  |  |  |  |  |  |  |
| Negative | 3 | 15.8 | 262 | 14.1 | 0.74 | 1.14 | 0.33–3.94 |
| Positive | 16 | 84.2 | 1594 | 85.9 |  |  |  |
| PR status |  |  |  |  |  |  |  |
| Negative | 6 | 31.6 | 419 | 22.8 | 0.41 | 1.56 | 0.59–4.14 |
| Positive | 13 | 68.4 | 1419 | 77.2 |  |  |  |
| HER2 status |  |  |  |  |  |  |  |
| Negative | 17 | 89.5 | 1519 | 85.5 | 1 | 1.44 | 0.33–6.26 |
| Positive | 2 | 10.5 | 257 | 14.5 |  |  |  |
| Grade |  |  |  |  |  |  |  |
| 1 (well diff.) | 3 | 15.0 | 359 | 19.0 | 0.48 | 1.59 | 0.57–4.38 |
| 2 (mod. diff.) | 12 | 60.0 | 876 | 46.4 | (1 and 2 vs. 3) |  |  |
| 3 (poorly diff.) | 5 | 25.0 | 653 | 34.6 |  |  |  |
| Morphology |  |  |  |  |  |  |  |
| Ductal | 15 | 75.0 | 1527 | 78.3 | 0.06 | 5.59 | 1.25–24.95 |
| Lobular | 2 | 10.0 | 295 | 15.1 | (Tubular vs. all other) |  |  |
| Tubular | 2 | 10.0 | 38 | 1.9 |  |  |  |
| Other^a^ | 1 | 5.0 | 90 | 4.6 |  |  |  |
| Type |  |  |  |  |  |  |  |
| Luminal A | 14 | 73.7 | 1353 | 76.6 | 0.24 | 1.92 | 0.55–6.67 |
| Luminal B | 2 | 10.5 | 183 | 10.4 | (Triple negative vs. all other) |  |  |
| HER2 | 0 | 0 | 74 | 4.2 |  |  |  |
| Triple negative | 3 | 15.8 | 157 | 8.9 |  |  |  |
| KI-67 |  |  |  |  |  |  |  |
| 0 | 2 | 10.5 | 141 | 7.9 | 0.11 | 2.30 | 0.87–6.08 |
| 1 | 11 | 57.9 | 725 | 40.6 | (0 and 1 vs. 2 and 3) |  |  |
| 2 | 2 | 10.5 | 480 | 26.9 |  |  |  |
| 3 | 4 | 21.1 | 440 | 24.6 |  |  |  |

CI=confidence interval; ER=estrogen receptor; M=primary metastasis; Mut=variant carrier; OR=odds ratio; PR=progesterone receptor; T=tumor size; WT=wild-type.

^a^ Includes medullary, mucinous, apocrine, papillary and neuroendocrine tumors

^b^ Fisher’s exact test

**Table C. Family history of cancers of *RAD52* delins positive index cases**

| Index ID -Cancers/tumors (age at diagnosis) | Breast/Ovarian cancer(s) in 1^st^ and/or 2^nd^ degree relatives (age at diagnosis if known) | Other cancers in 1^st^ and/or 2^nd^ degree relatives (age at diagnosis if known) |
| --- | --- | --- |
| Her1 -BC (36)^d^ | - | Leu (9), Sto (41), Lung (60), CSU, CSU |
| Her2 -Bil BC (34)^d^ | BC | Ut [+], Sto [+], Sgt + Lym [+], Lung (71), CSU (59), Sgt [-] |
| Her3 -BC (54)^d^ | BC (36) | CSU (30) |
| Her4 -BC (78) + Skin (73)^c^ | BC (50) + Cho (64) [+] | Pro |
| Her5 -BC (26)^c^ | BC (62) [+], BC (61) | - |
| Her6 -BC (28)^a,d^ | - | - |
| Her7 -BC (47)^b,c^ | BC (64) + Bas [+], BC (50) [+], BC | Pan (50) [+] |
| Uns1 -BC (50)^b,d^ | BC (57), BC (64), BC (76) + Lym (88) | - |
| Uns2 -BC (61)^d^ | - | - |
| Uns3 -BC (54)^d^ | BC (65) | Lung (62), Col + Hep + Lym, Pan |
| Uns4 -Bil BC (71,75)^d^ | - | - |
| Uns5 -BC (53)^d^ | BC | CSU |
| Uns6 -BC (63)^d^ | - | - |
| Uns7 -BC (78) + Ut (78)^d^ | - | Mye |
| Uns8 -BC (71)^d^ | BC (63) | - |
| Uns9 -BC (75)^d^ | - | - |
| Uns10 -BC (64)^d^ | - | - |
| Uns11 -BC (60)^e^ | BC (54) [-], BC (80), BC | Thy (13), Wil (1), Lar (67), Bone |
| Uns12 -BC (52)^d^ | - |  |
| Uns13 -BC (50)^d^ | - | Lung (64) |
| Uns14 -BC (58)^d^ | - | Pan (76) |
| Uns15 -BC (60)^d^ | - | - |
| Uns16 -BC (50)^d^ | - | - |
| Uns17 -Bil BC (59)^d^ | - | - |
| Uns18 -BC (48)^d^ | BC (88) | Cer (63) [-], Lung (50) |
| Uns19 -BC (63)^d^ | BC (48) | - |
| Uns20 -BC (73)^d^ | - | Sto |

- : none reported; Her=hereditary cohort; Uns=unselected cohort; BC=breast cancer; Bil BC=bilateral breast cancer; Bas=basalioma; Cer=cervical cancer; Cho=chondrosarcoma; Col=colorectal cancer; Hep=hepatic cancer; Lar=laryngeal cancer; Leu=leukemia; Lym=lymphoma; Mye=myeloma; Pan=pancreatic cancer; Pro=prostate cancer; Sgt=salivary gland tumor; Sto=stomach cancer; Thy=thyroid cancer; Ut=uterine cancer; Wil=Wilms’ tumor; CSU=cancer site unknown.

All tested cases marked as [+], if positive and [-], if negative for *RAD52* delins.

^a^ Carrier of *RAD52* stop-gain variant p.Tyr415Ter (rs4987208) and *RAD51C* duplication

^b^ Carrier of *RAD52* stop-gain variant p.Ser346Ter

^c^ Supporting, ^d^ inconclusive or ^e^ no supporting evidence for delins allele co-segregating with breast cancer in the family

**Table D.** **Frequency of *RAD52*** **p.Ser346Ter (rs4987207) and p.Tyr415Ter (rs4987208) in the studied breast cancer cases and controls**

| Cohort | N | WT | % | Mut^c^ | % | OR | 95% CI | P^d^ |
| --- | --- | --- | --- | --- | --- | --- | --- | --- |
| *RAD52* p.Ser346Ter |  |  |  |  |  |  |  |  |
| Hereditary BC^a^ | 278 | 264 | 95.0 | 14 | 5.0 | 1.90 | 1.01–3.58 | 0.06 |
| Discovery | 98 | 92 | 93.9 | 6 | 6.1 |  |  |  |
| Replication | 180 | 172 | 95.6 | 8 | 4.4 |  |  |  |
| Unselected BC | 1983 | 1932 | 97.4 | 51 | 2.6 | 0.95 | 0.61–1.46 | 0.83 |
| All BC | 2261 | 2196 | 97.1 | 65 | 2.9 | 1.06 | 0.70–1.60 | 0.84 |
| Controls^b^ | 1327 | 1291 | 97.3 | 36 | 2.7 |  |  |  |
| *RAD52* p.Tyr415Ter |  |  |  |  |  |  |  |  |
| Hereditary BC^a^ | 278 | 268 | 96.4 | 10 | 3.6 | 1.09 | 0.54–2.19 | 0.86 |
| Discovery | 98 | 95 | 96.9 | 3 | 3.1 |  |  |  |
| Replication | 180 | 173 | 96.1 | 7 | 3.9 |  |  |  |
| Unselected BC | 1983 | 1927 | 97.2 | 56 | 2.8 | 0.85 | 0.57–1.27 | 0.47 |
| All BC | 2261 | 2195 | 97.1 | 66 | 2.9 | 0.88 | 0.60–1.29 | 0.55 |
| Controls^b^ | 1327 | 1283 | 96.7 | 44 | 3.3 |  |  |  |

BC=breast cancer; CI=confidence interval; Mut=variant carrier; OR=odds ratio; WT=wild-type.

^a^ Combined Discovery and Replication cohort

^b^ Frequency in the general population in Northern Finland obtained from SISu

^c^ All heterozygous

^d^ Fisher’s exact test, respective case cohort (Hereditary, Unselected, All BC) vs. Controls

**Table E. Tumor parameters of unselected cohort *HSD17B14* deletion carriers compared with the tumors of non‐carriers**

| Category | Mut | % | WT | % | P^b^ | OR | 95% CI |
| --- | --- | --- | --- | --- | --- | --- | --- |
| T class |  |  |  |  |  |  |  |
| 1 | 12 | 60.0 | 1089 | 64.1 | 0.82 | 0.84 | 0.34–2.07 |
| 2 | 8 | 40.0 | 534 | 31.4 | (1 vs. 2, 3, 4) |  |  |
| 3 | 0 | 0 | 55 | 3.2 |  |  |  |
| 4 | 0 | 0 | 21 | 1.2 |  |  |  |
| M class |  |  |  |  |  |  |  |
| Negative | 22 | 100.0 | 1933 | 97.8 | 1 | - | - |
| Positive | 0 | 0 | 44 | 2.2 |  |  |  |
| Node status |  |  |  |  |  |  |  |
| Negative | 13 | 59.1 | 1276 | 64.9 | 0.65 | 0.78 | 0.33–1.84 |
| Positive | 9 | 40.9 | 691 | 35.1 |  |  |  |
| ER status |  |  |  |  |  |  |  |
| Negative | 1 | 4.5 | 264 | 14.2 | 0.35 | 0.29 | 0.04–2.14 |
| Positive | 21 | 95.5 | 1589 | 85.8 |  |  |  |
| PR status |  |  |  |  |  |  |  |
| Negative | 2 | 9.1 | 423 | 23.1 | 0.12 | 0.33 | 0.08–1.43 |
| Positive | 20 | 90.9 | 1412 | 76.9 |  |  |  |
| HER2 status |  |  |  |  |  |  |  |
| Negative | 22 | 100.0 | 1514 | 85.4 | 0.06 | - | - |
| Positive | 0 | 0 | 259 | 14.6 |  |  |  |
| Grade |  |  |  |  |  |  |  |
| 1 (well diff.) | 4 | 18.2 | 358 | 19.0 | 0.37 | 1.80 | 0.66–4.90 |
| 2 (mod. diff.) | 13 | 59.1 | 875 | 46.4 | (1 and 2 vs. 3) |  |  |
| 3 (poorly diff.) | 5 | 22.7 | 653 | 34.6 |  |  |  |
| Morphology |  |  |  |  |  |  |  |
| Ductal | 21 | 91.3 | 1521 | 78.1 | 0.20 | 2.94 | 0.69–12.59 |
| Lobular | 1 | 4.3 | 296 | 15.2 | (Ductal vs. all other) |  |  |
| Other^a^ | 1 | 4.3 | 130 | 6.7 |  |  |  |
| Type |  |  |  |  |  |  |  |
| Luminal A | 21 | 95.5 | 1346 | 76.3 | 0.04 | 6.52 | 0.88–48.63 |
| Luminal B | 0 | 0 | 185 | 10.5 | (Luminal A vs. all other) |  |  |
| HER2 | 0 | 0 | 74 | 4.2 |  |  |  |
| Triple negative | 1 | 4.5 | 159 | 9.0 |  |  |  |
| KI-67 |  |  |  |  |  |  |  |
| 0 | 0 | 0 | 143 | 8.0 | 1 | 1.05 | 0.46–2.44 |
| 1 | 11 | 50.0 | 725 | 40.7 | (0 and 1 vs. 2 and 3) |  |  |
| 2 | 5 | 22.7 | 477 | 26.8 |  |  |  |
| 3 | 6 | 27.3 | 438 | 24.6 |  |  |  |

CI=confidence interval; ER=estrogen receptor; M=primary metastasis; Mut=variant carrier; OR=odds ratio; PR=progesterone receptor; T=tumor size; WT=wild-type.
^a^ Includes medullary, tubular, mucinous, apocrine, papillary and neuroendocrine tumors ^b^ Fisher’s exact test

**Table F.** **Menopausal status of unselected cohort *HSD17B14* deletion carriers compared with the menopausal status of non-carriers**

|  | Mut |  | % | WT | % | p^a^ | OR | 95% CI |
| --- | --- | --- | --- | --- | --- | --- | --- | --- |
| Menopause | 24 |  |  | 1959 |  | 0.16 | 2.44 | 0.73–8.23 |
| Pre | 3 |  | 12.5 | 507 | 25.9 | (Post vs. Pre) |  |  |
| Post | 21 |  | 87.5 | 1452 | 74.1 |  |  |  |

CI=confidence interval; Mut=variant carrier; OR=odds ratio; WT=wild-type.
^a^ Fisher’s exact test

**Table G. Family history of cancers of *HSD17B14* deletion positive index cases**

| Index ID -Cancers/tumors (age at diagnosis) | Breast/Ovarian cancer(s) in 1^st^ and/or 2^nd^ degree relatives (age at diagnosis if known) | Other cancers in 1^st^ and/or 2^nd^ degree relatives (age at diagnosis if known) |
| --- | --- | --- |
| Her8 -Bil BC (39, 45)^d^ | - | Leu (60), Hep (60) |
| Her9 -BC (36)^d^ | - | - |
| Her10 -BC (51)^c^ | BC (59) + Bas (78) + Mel (78) [+]^a^, BC (50) [+]^a^, BC | Thy (47) [+] |
| Her11 -Bil BC (40)^c^ | BC (42) [+] | Pan [+], Ut (36) [+], Lung, Lung |
| Her12 -BC (45)^d^ | BC (47) [+], BC (41) [-], BC (55) | Col (43) [-] |
| Her13 -Bil BC (37) + Bas (52)^d^ | BC + Ov, BC (52) | Mel (81) [-] |
| Her14 -BC (56) + Pv (62)^d^ | Ov [+], BC (69) + Ut (51) [-] | Thy (63), Eye mel (26), Pro (80), Oe (82) |
| Her15 -BC (28)^d^ | BC, BC | Hep |
| Uns21 -BC (47)^d^ | BC (57), BC (50) | Ren (60) |
| Uns22 -BC (62)^d^ | - | Pro + Lung, Lung, Pan (79) |
| Uns23 -BC (80)^d^ | BC | Lung |
| Uns24 -BC (61)^e^ | BC (54) + Gall (56) + Bas (61) [-], BC (60) | Mel (70) |
| Uns25 -BC (60)^e^ | BC (68) [-] | Pro (70), Pan (78), Oe (66) |
| Uns26 -BC (54)^d^ | BC, BC | Lung, Lung |
| Uns27 -BC (68)^d^ | - | - |
| Uns28 -BC (55)^d^ |  | CSU (84) |
| Uns29 -BC (63)^d^ | - |  |
| Uns30 -BC (58) + Bas (69)^d^ | - | Lym (61), Oe, Lung |
| Uns31 -BC (57)^d^ | - | - |
| Uns32 -BC (52)^d^ | - | Cer (68) + Pv (70) [+], Thy (67) |
| Uns33 -BC (63)^d^ | - | Lung (62) |
| Uns34 -BC (59)^d^ | BC | Pro (75) [+] |
| Uns35 -BC (70)^d^ | BC (54), BC | - |
| Uns36 -BC (72)^d^ | - | Eye mel (60), Lung, Sto, Sto |
| Uns37 -BC (61)^d^ | - | Pro, CSU (70) |
| Uns38 -BC (65)^b,d^ | - |  |
| Uns39 -Bil BC (47, 52)^d^ | - | - |
| Uns40 -BC (47)^d^ | - | - |
| Uns41 -BC (53)^d^ | - |  |
| Uns42 -BC (74)^d^ | - | Sto (48), Ut (49), Brain (7) |
| Uns43 -BC (76)^d^ | - | - |
| Uns44 -BC (53)^c^ | BC (45, 80) + Bas (80) [+], BC (71) [+], BC | Leu (33), Col (73) + Skin (73) [+] |

- : none reported; Her=hereditary cohort; Uns=unselected cohort; BC=breast cancer; Bil BC=bilateral breast cancer; Bas=basalioma; Cer=cervical cancer; Col=colorectal cancer; CSU=cancer site unknown; Eye mel=eye melanoma; Gall=gallbladder cancer; Hep=hepatic cancer; Leu=leukemia; Lym=lymphoma; Mye=myeloma; Mel=melanoma; Oe=oesophageal cancer; Ov=ovarian cancer; Pan=pancreatic cancer; Pv=polycythemia vera; Ren=renal cancer; Sgt=salivary gland tumor; Sto=stomach cancer; Thy=thyroid cancer; Ut=uterine cancer.

All tested cases marked as [+], if positive and [-], if negative for *HSD17B14* deletion.

^a^ Carrier of pathogenic *ATM* c.7570G>C (p.Ala2524Pro)

^b^ Carrier of *RAD51C* duplication

^c^ Supporting, ^d^ inconclusive or ^e^ no supporting evidence for deletion allele co-segregating with breast cancer in the family

**Table H. Tumor parameters of unselected cohort *RAD51C* duplication carriers compared with the tumors of non‐carriers**

| Category | Mut | % | WT | % | P^b^ | OR | 95% CI |
| --- | --- | --- | --- | --- | --- | --- | --- |
| T class |  |  |  |  |  |  |  |
| 1 | 4 | 66.7 | 1097 | 64.0 | 1 | 1.12 | 0.21–6.15 |
| 2 | 1 | 16.7 | 541 | 31.6 | (1 vs. 2, 3, 4) |  |  |
| 3 | 1 | 16.7 | 54 | 3.1 |  |  |  |
| 4 | 0 | 0 | 21 | 1.2 |  |  |  |
| M class |  |  |  |  |  |  |  |
| Negative | 5 | 83.3 | 1929 | 97.8 | 0.13 | 8.97 | 1.03–78.44 |
| Positive | 1 | 16.7 | 43 | 2.2 |  |  |  |
| Node status |  |  |  |  |  |  |  |
| Negative | 3 | 50.0 | 1272 | 64.8 | 0.43 | 1.84 | 0.37–9.16 |
| Positive | 3 | 50.0 | 690 | 35.2 |  |  |  |
| ER status |  |  |  |  |  |  |  |
| Negative | 0 | 0 | 265 | 14.2 | 1 | - | - |
| Positive | 6 | 100.0 | 1604 | 85.8 |  |  |  |
| PR status |  |  |  |  |  |  |  |
| Negative | 0 | 0 | 425 | 23.0 | 0.35 | - | - |
| Positive | 6 | 100.0 | 1426 | 77.0 |  |  |  |
| HER2 status |  |  |  |  |  |  |  |
| Negative | 5 | 83.3 | 1531 | 85.6 | 1 | 0.84 | 0.10–7.24 |
| Positive | 1 | 16.7 | 258 | 14.4 |  |  |  |
| Grade |  |  |  |  |  |  |  |
| 1 (well diff.) | 0 | 0 | 362 | 19.0 | 0.42 | 0.53 | 0.11–2.61 |
| 2 (mod. Diff.) | 3 | 50.0 | 885 | 46.5 | (1 and 2 vs. 3) |  |  |
| 3 (poorly diff.) | 3 | 50.0 | 655 | 34.4 |  |  |  |
| Morphology |  |  |  |  |  |  |  |
| Ductal | 4 | 66.7 | 1538 | 78.3 | 0.23 | 2.83 | 0.52–15.51 |
| Lobular | 2 | 33.3 | 295 | 15.0 | (Lobular vs. all other) |  |  |
| Other^a^ | 0 | 0 | 131 | 6.7 |  |  |  |
| Type |  |  |  |  |  |  |  |
| Luminal A | 5 | 83.3 | 1362 | 76.5 | 1 | 1.54 | 0.18–13.17 |
| Luminal B | 1 | 16.7 | 184 | 10.3 | (Luminal A vs. all other) |  |  |
| HER2 | 0 | 0 | 74 | 4.1 |  |  |  |
| Triple negative | 0 | 0 | 160 | 9.0 |  |  |  |
| KI-67 |  |  |  |  |  |  |  |
| 0 | 0 | 0 | 143 | 7.9 | 0.69 | 0.53 | 0.10–2.88 |
| 1 | 2 | 33.3 | 734 | 40.8 | (0 and 1 vs. 2 and 3) |  |  |
| 2 | 0 | 0 | 482 | 26.8 |  |  |  |
| 3 | 4 | 66.7 | 440 | 24.5 |  |  |  |

CI=confidence interval; ER=estrogen receptor; M=primary metastasis; Mut=variant carrier; OR=odds ratio; PR=progesterone receptor; T=tumor size; WT=wild-type.

^a^ Includes medullary, tubular, mucinous, apocrine, papillary and neuroendocrine tumors ^b^ Fisher’s exact test

**Table I. Family history of cancers of *RAD51C* duplication positive index cases**

| Index ID -Cancers/tumors (age at diagnosis) | Breast/Ovarian cancer(s) in 1^st^ and/or 2^nd^ degree relatives (age at diagnosis if known) | Other cancers in 1^st^ and/or 2^nd^ degree relatives (age at diagnosis if known) |
| --- | --- | --- |
| Her16 -BC (44)^a,f^ | BC (58) [-], BC (51) + Endo (54) [-], BC (70), BC + Ov | Lung, Pro (76), Pro (55), Pro (71) + Bla (71), Brain (55), Sto (50), CSU |
| Her6 -BC (28)^b,e^ | - | - |
| Uns45 -BC (53)^e^ | BC, BC | - |
| Uns46 -BC (46)^e^ | BC (56), BC | Sto (29), CSU (55), CSU, CSU |
| Uns47 -BC (80)^e^ | - | - |
| Uns48 -BC (39)^e^ | - | Leu (45) |
| Uns38 -BC (65)^c,e^ | - | - |
| Uns49 -BC (79)^e^ | - | - |

- : none reported; Her=hereditary cohort; Uns=unselected cohort; BC=breast cancer; Bla=bladder cancer; CSU=cancer site unknown; Endo=endometrial cancer; Leu=leukemia; Ov=ovarian cancer; Pan=pancreatic cancer; Pro=prostate cancer; Sto=stomach cancer.

All tested cases marked as [+], if positive and [-], if negative for *RAD51C* duplication

^a^ Carrier of pathogenic *ATM* c.7570G>C (p.Ala2524Pro)

^b^ Carrier of *RAD52* delins and *RAD52* stop-gain variant (rs4987208)

^c^ Carrier of *HSD17B14* deletion

^d^ Supporting, ^e^ inconclusive or ^f^ no supporting evidence for duplication allele co-segregating with breast cancer in the family
